# Bayesian-Optimized Convolutional Neural Networks for Classifying Primary Tumor Origin of Brain Metastases from MRI

**DOI:** 10.3390/brainsci15050450

**Published:** 2025-04-25

**Authors:** Jawed Nawabi, Semil Eminovic, Alexander Hartenstein, Georg Lukas Baumgaertner, Nils Schnurbusch, Madhuri Rudolph, David Wasilewski, Julia Onken, Eberhard Siebert, Edzard Wiener, Georg Bohner, Andrea Dell’Orco, Mike P. Wattjes, Bernd Hamm, Uli Fehrenbach, Tobias Penzkofer

**Affiliations:** 1 Department of Neuroradiology, Charité—Universitätsmedizin, 10117 Berlin, Germany; jawed.nawabi@charite.de (J.N.); eberhard.siebert@charite.de (E.S.); edzard.wiener@charite.de (E.W.); georg.bohner@charite.de (G.B.); andrea.dellorco@charite.de (A.D.); mike.wattjes@charite.de (M.P.W.); 2 Department of Radiology, Charité—Universitätsmedizin, 10117 Berlin, Germany; georg.baumgaertner@charite.de (G.L.B.); nils.schnurbusch@charite.de (N.S.); madhuri.rudolph@charite.de (M.R.); bernd.hamm@charite.de (B.H.); uli.fehrenbach@charite.de (U.F.); tobias.penzkofer@charite.de (T.P.); 3 Medical Affairs and Pharmacovigilance, Bayer AG, 13353 Berlin, Germany; ahartens@gmail.com; 4 Department of Neurosurgery, Medical Faculty & University Hospital Düsseldorf, Heinrich Heine University Dusseldorf, 40225 Dusseldorf, Germany; davidwasilewski2@gmail.com; 5 Department of Neurosurgery, Charité—Universitätsmedizin, 10117 Berlin, Germany; julia.onken@charite.de; 6 Berlin Institute of Health (BIH), 10117 Berlin, Germany

**Keywords:** brain metastases, neural networks, Bayesian optimization

## Abstract

**Background/Objectives**: This study evaluates whether convolutional neural networks (CNNs) can be trained to determine the primary tumor origin from MRI images alone in patients with metastatic brain lesions. **Methods**: This retrospective, monocentric study involved the segmentation of 1175 brain lesions from MRI scans of 436 patients with histologically confirmed primary tumor origins. The four most common tumor types—lung adenocarcinoma, small cell lung cancer, breast cancer, and melanoma—were selected, and a class-balanced dataset was created through under-sampling. This resulted in 276 training datasets and 88 hold-out test datasets. Bayesian optimization was employed to determine the optimal CNN architecture, the most relevant imaging sequences, and whether the masking of images was necessary. We compared the performance of the CNN with that of two expert radiologists specializing in neuro-oncological imaging. **Results**: The best-performing CNN from the Bayesian optimization process used masked images across all available MRI sequences. It achieved Area-Under-the-Curve (AUC) values of 0.75 for melanoma, 0.65 for small cell lung cancer, 0.64 for breast cancer, and 0.57 for lung adenocarcinoma. Masked images likely improved performance by focusing the CNN on relevant regions and reducing noise from surrounding tissues. In comparison, Radiologist 1 achieved AUCs of 0.55, 0.52, 0.45, and 0.51, and Radiologist 2 achieved AUCs of 0.68, 0.55, 0.64, and 0.43 for the same tumor types, respectively. The CNN consistently showed higher accuracy, particularly for melanoma and breast cancer. **Conclusions**: Bayesian optimization enabled the creation of a CNN that outperformed expert radiologists in classifying the primary tumor origin of brain metastases from MRI.

## 1. Introduction

Brain metastases are the most common intracranial tumors in adults, often occurring in an advanced stage of systemic cancer [1]. An estimated 9 to 50% of patients with malignant cancer develop brain metastases [2]. Lung cancer, breast cancer, and melanoma, which account for 67–87% of all cancers, are the most frequent to develop brain metastases [3]. However, the primary cancer is unknown in 15% of patients presenting with occult brain metastases [4].

Accurate identification of tumor etiology in patients with occult brain metastases is crucial for guiding appropriate therapeutic strategies [5,6]. Histopathological analysis following biopsy or resection is the gold standard for determining the primary source of metastasis [5]. In addition, non-invasive approaches, such as magnetic resonance imaging (MRI), have become increasingly important for diagnosis [7,8], and disease staging, especially when tissue sampling is impractical or poses risks. Recent advancements in artificial intelligence (AI), particularly convolutional neural networks (CNNs), have shown substantial promise in automating the analysis of medical imaging data. These models can enhance diagnostic accuracy and reduce reliance on invasive procedures by capturing complex spatial features in imaging data [9,10,11,12,13]. CNNs, along with radiomics-driven machine learning models, have become central tools in brain metastases analysis [14,15,16]. However, despite encouraging progress [17,18,19], several key limitations persist in the application of deep learning models for classifying the etiology of brain metastases.

Firstly, CNNs often require extensive labeled datasets to generalize effectively. In the clinical context, annotated brain metastases datasets are relatively limited due to privacy constraints, labor-intensive labeling, and the rarity of certain metastatic origins. This data scarcity leads to overfitting and poor generalization on unseen cases. Secondly, there is significant heterogeneity in tumor presentation on MRI. Tumors may differ in size, location, signal intensity, and morphology, which poses a challenge for CNNs that lack mechanisms for uncertainty estimation or robust feature selection.

Furthermore, most conventional CNN architectures rely on trial-and-error or grid [20]/random search strategies for hyperparameter tuning. These methods are computationally inefficient and may converge on suboptimal models. Even when using advanced architectures like ResNet or U-Net, performance is highly sensitive to design choices such as learning rates, number of layers, or convolutional filter sizes. Moreover, integrating multimodal data (e.g., clinical features with radiomic data [21]) has shown potential for performance gains, but this adds complexity to the model design and tuning process.

Importantly, current deep learning pipelines rarely account for uncertainty in model predictions—a critical consideration in clinical settings where misclassification can lead to inappropriate treatment choices. This lack of uncertainty quantification also limits trust and interpretability in AI-assisted diagnosis.

To address these limitations, Bayesian optimization presents a promising alternative. Bayesian optimization is an efficient global optimization technique that can automate the search for high-performing hyperparameters or model configurations using a probabilistic surrogate model [22]—by guiding the search for optimal parameters (e.g., learning rates, layer configurations), it can improve model performance and training efficiency in MRI classification [23]. It not only accelerates the development cycle but also helps avoid biased or arbitrary model design. By integrating Bayesian optimization into CNN training, we can systematically explore the parameter space to construct models that generalize better and perform more reliably on complex imaging tasks.

The aim of this study was to develop a convolutional neural network (CNN) using Bayesian optimization to guide neural network architecture design and input data selection without bias. We hypothesize that this CNN can accurately predict the primary tumor origin of brain metastases from MRI scans, providing a non-invasive diagnostic tool to assist clinicians in determining tumor etiology and optimizing treatment strategies. By leveraging a dataset of MRI images with known metastatic origins, we aim to assess the feasibility and clinical applicability of this deep learning approach.

## 2. Materials and Methods

### 2.1. Study Cohort

The study was approved by the Charité Ethics Committee [EA2/033/18], and due to the retrospective design, the need for informed written consent was waived in accordance with institutional guidelines and regulations. The study was performed in accordance with the Declaration of Helsinki.

**Figure 1:**
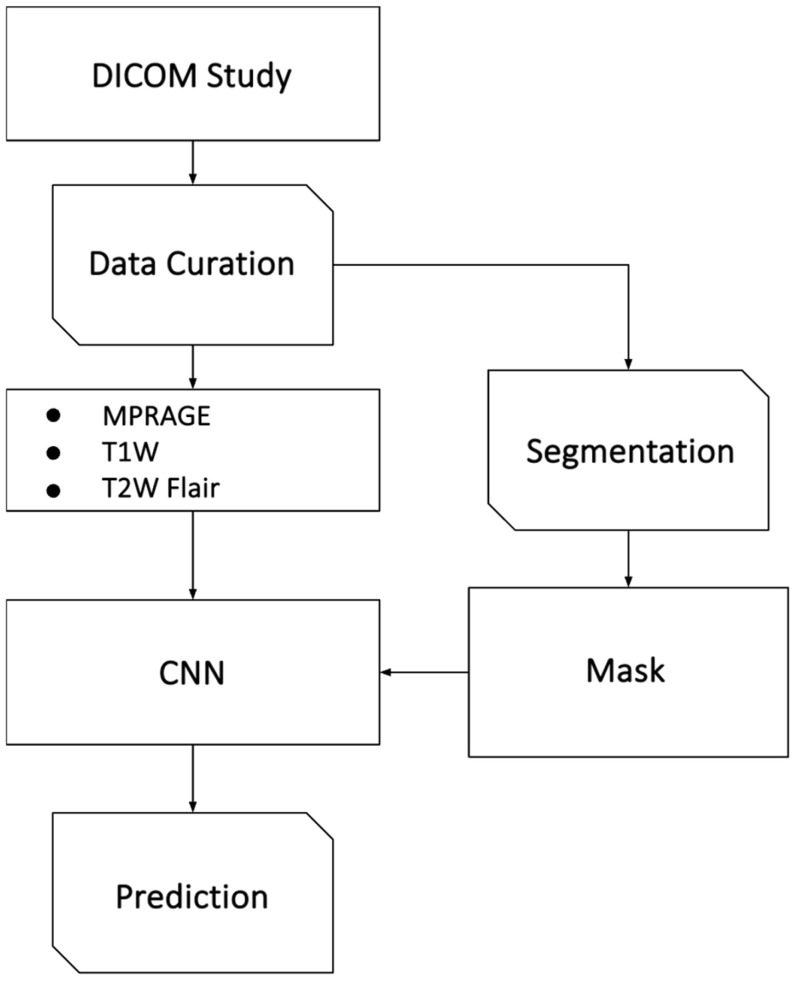
Overview of the study workflow. This figure illustrates the methodological pipeline used in the study. The process begins with patient selection based on defined inclusion and exclusion criteria, followed by retrospective MRI data curation (MRI sequences MPRAGE, T1w, T2w FLAIR). Imaging data were preprocessed via atlas registration, bias correction, and normalization. Metastatic lesions were segmented and labeled based on histopathology. Images were processed either without segmentation mask information, as masked images (with values outside the segmentation set to zero), or with the segmentation mask provided as a separate channel alongside the image modalities. Data are split into training, validation, and test sets, with augmentation applied during training. A CNN is optimized using Bayesian optimization and evaluated on a test set.

In this retrospective study, the inclusion criteria were as follows: Brain MRI performed on a 1.5 T whole body MRI system (Magnetom Aera, Siemens Healthcare, Erlangen, Germany) between May 2012 and January 2018, including 3D T1weighted (w) MPRAGE post-contrast-enhanced (CE), axial T1w TSE post-contrast, and T2w FLAIR sequences; the presence of metastatic brain lesions; and a histopathologically confirmed primary cancer. Exclusion criteria included insufficient imaging quality, absence of metastatic brain lesions, presence of primary brain tumors, and absence of the selected imaging sequences. A total of 436 patients met the inclusion criteria and were included in the final dataset. To mitigate overfitting and bias, we utilized only one MRI series per patient. Our overall study workflow is illustrated in Figure 1.

### 2.2. Imaging Analysis

Brain MRI scans were obtained from the local PACS archive, anonymized, and converted to NIFTI format for further analysis. Metastatic lesions were independently reviewed and rated by two expert neuroradiologists, each with more than 10 years of expertise in neuro-oncological imaging. In cases of discrepancies between the two radiologists, a consensus reading was conducted to resolve any differences. Metastatic lesions were semi-automatically segmented using the MITK software suite (MITK v. 2016.3.0, DKFZ, Heidelberg, Germany) [24]. A total of 1175 metastatic tumors were semi-automatically segmented from the patient cohort. Metastatic lesions were assigned a tumor entity label based on the histopathology of the primary tumor. The possible labels included Lung Adenocarcinoma, Squamous Cell Lung Carcinoma, Small Cell Lung Cancer, Melanoma, Breast Cancer, and Undefined Lung Cancer. However, for the final datasets used in training, testing, and validation, only four tumor classes—Lung Adenocarcinoma, Small Cell Lung Cancer, Melanoma, and Breast Cancer—were included.

### 2.3. Model Training, Optimization, and Performance Evaluation

To address class imbalance, a 50:50 class balancing technique was applied using under-sampling; 15% of the datasets from each class were reserved for testing, namely 22 image datasets per class for a total of 88 image datasets, were set aside for testing. The remaining datasets were used for training and validation.

The image postprocessing pipeline included atlas registration, N4 bias correction, and white stripe normalization. First, T1w TSE post-contrast images were registered to the Montreal Neurological Institute (MNI) 152 atlases Brain Atlas [25] using the NiftyReg reg_aladin function [26]. The output affine matrix was applied to transform both T1w MPRAGE post-contrast and T2w FLAIR imaging sequences, which were resampled to 0.5 × 0.5 × 0.5 to prevent step artifacts. Following atlas registration, images were N4 bias-corrected using N4ITK [27]. For whitestripe normalization, whitestripe masks were generated using skull-stripped T1w TSE post-CE images; skull stripping was performed using the BET2 software tool (v. FSL 6.0.4). These masks were then applied on all imaging sequences using the WhiteStripe library in R (v. 4.0.5) [28]. Finally, images were resampled to an isotropic resolution of 1 × 1 × 1 mm^3^. A final volume of 80 × 80 × 80 mm^3^ was cropped around each tumor segmentation. Online image augmentation was performed during training only, involving four augmentations: Brightness variation (0.5–1.5), rotation (±90 degrees), translation (up to 10 voxels in x,y,z axis), and flipping across the sagittal and/or axial plane. Afterward, images were cropped to a final volume of 48 × 48 × 48 mm^3^ before being fed to the neural network. Four-fold cross-validation was performed, resulting in the training of four neural networks, each using a different set of 204 image datasets for training and 72 for validation, corresponding to distinct hyperparameter sets.

In this study, we implemented Bayesian optimization, a sequential model-based optimization (SMBO) technique, to optimize our neural network architecture and hyperparameters for image classification tasks. Bayesian optimization is particularly well-suited for black-box functions where derivatives are unavailable or difficult to compute, making it ideal for tuning complex machine learning models [29,30]. Additional details on the SMBO method are available in the Supplementary Materials.

In our study, Bayesian optimization was employed to guide the selection of key hyperparameters, including the input image modality, masking procedure, and neural network architecture for the CNN. This approach allowed us to efficiently explore the hyperparameter space and balance exploration with optimization, ensuring that the final model configuration would yield optimal performance on MRI-based classification tasks. The input image types included any single image sequence or a combination of the T1w MPRAGE post-CE, T1w TSE post-CE, and T2w FLAIR. Images were processed either without segmentation mask information, as masked images (with values outside the segmentation set to zero), or with the segmentation mask provided as a separate channel alongside the image modalities. The CNN architecture consisted of a series of convolutional layers followed by a series of fully connected layers, with specific hyperparameters determined through Bayesian optimization. The convolutional layers were grouped into [2,3] ‘convolutional segments’, each containing [1,2,3,4] convolutional layers. Within each segment, the layers shared A kernel size of [3, 5] in the x,y direction and [1, 3] in z, with an output depth of [8, 64, 128]. Each convolutional segment was followed by a max-pooling layer. The output from the convolutional layers was flattened and passed through [1,2,3,4,5,6] fully connected layers with [512, 1024, 1536, 2048] neurons, culminating in a final connected layer of 128 neurons.

Adam optimization was used to update network weights [31], with parameters set as follows: Alpha, beta1, beta2 and epsilon set to 0.0001, 0.9, 0.999 and 1 × 10^−8^ [31]. All models were implemented using Keras and Tensorflow (v. 1.10.1) and executed on an Nvidia Quadro GV100 graphics card. Bayesian optimization was performed using the Hyperopt package in Python (v. 3.8.10) [32]. The loss function was defined as one minus the area under the Receiver Operating Characteristics (ROC) curve, averaged across the four output classes and further averaged over the four folds of k-fold validation. After 100 iterations of Bayesian optimization, the highest-performing set of hyperparameters was used to train a final neural network that took all 276 available training images without a separate validation set. This final model was then tested on a test dataset of 88 images.

For performance comparison, the CNN’s performance was evaluated against the diagnoses of two expert neuro-oncological radiologists, each with more than 10 years of expertise in neuro-oncological imaging (U.F., Radiologist 1; E.S., Radiologist 2), who independently analyzed T1w MPRAGE post-contrast, T1w TSE post-contrast, and T2w FLAIR images to classify the brain metastases by the primary tumor entity. To benchmark its performance, the Area-Under-the-Curve (AUC) values of the CNN were compared with those of two experienced radiologists. Additionally, confusion matrices were used to provide a detailed breakdown of true positives, false positives, true negatives, and false negatives across all tumor categories. Performance metrics, including AUC values and ROC curves, were calculated using the scikit-learn library in Python [33]. Confusion matrices were also generated and visualized as heatmaps using tools from the same library.

## 3. Results

Over the 100 variations of hyperparameters explored, the models achieving mean AUC values on the validation set ranging between 0.50 and 0.63 were created. The average training time per optimization step, which involved four-fold validation for each combination of hyperparameters, was 54 ± 42 min (SD), with a minimum training time of 9 min and maximum of 187 min per step. The total optimization process took 5379 min, equaling approximately 89.7 h.

**Figure 2:**
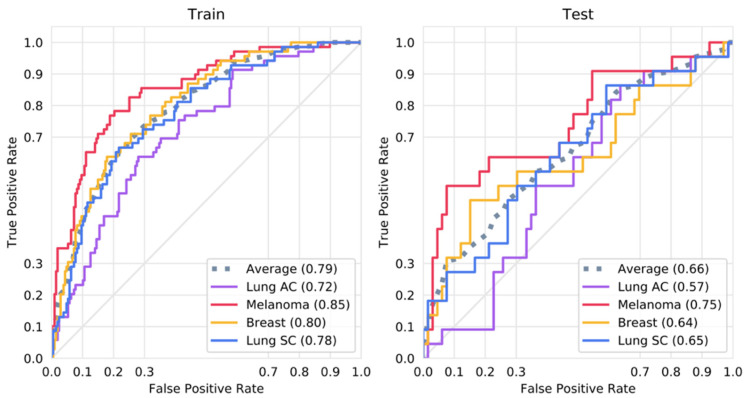
ROC curves for the training and test sets. Shown is the ROC curve for a CNN trained with all available training data using the optimal parameters as selected by Bayesian optimization on the training and test dataset.

The best-performing set of hyperparameters, as selected by Bayesian optimization, yielded a CNN with a mean AUC of 0.79 on the training sets and 0.66 on the validation sets, averaged across all four output classes. After retraining using the selected parameters on the entire training dataset, the CNN achieved AUCs of 0.75 for melanoma, 0.64 for small cell lung cancer, 0.64 for breast cancer, and 0.57 for lung adenocarcinoma on the test dataset. On the training set, it achieved AUCs of 0.85, 0.78, 0.80, and 0.72 for the same respective classes. The ROC curves for the training and test sets are shown in Figure 2.

**Figure 3:**
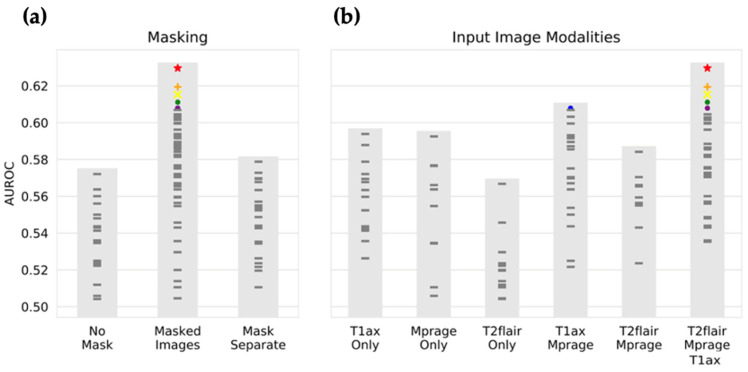
Input image selection by Bayesian optimization. The input image type that results in the highest performance is selected by Bayesian optimization. Each mark represents the performance of CNNs with a unique set of hyperparameters, as selected by Bayesian optimization. Shown on the y-axis is the area under the ROC curve on the validation set, averaged over the four output classes (breast cancer, small cell lung cancer, lung adenocarcinoma, and melanoma) and the four folds of k-fold validation. In (**a**), the selection of masking type is displayed, with the highest performance with an average AUC of 0.63, resulting from masked input images, and the lowest performance resulting from no segmentation mask being provided. In (**b**), the selection of input image modality is shown, with the best performance resulting from receiving all image modalities (T1w CE (contrast enhancement), T1w MPRAGE CE, and T2wFLAIR), and the worst performance on receiving only T2FLAIR images. Colored marks are the top seven performing neural networks. For instance, the red star is the highest-performing combination of hyperparameters, with Masked Images and T2w FLAIR, T1w MPRAGE CE, and T1w CE, while the blue mark took masked images and only T1w CE and T1w MPRAGE CE (no T2w FLAIR). *T1ax Only* refers to T1w CE; *Mprage Only* to T1w MPRAGE CE; *T2flair Only* to T2w FLAIR; *T1ax Mprage* to T1w CE and T1w MPRAGE CE; *T2flair Mprage* to T2w FLAIR and T1w MPRAGE CE; *T2flair Mprage T1ax* to T2w FLAIR, T1w MPRAGE CE, and T1w MPRAGE CE.

**Figure 4:**
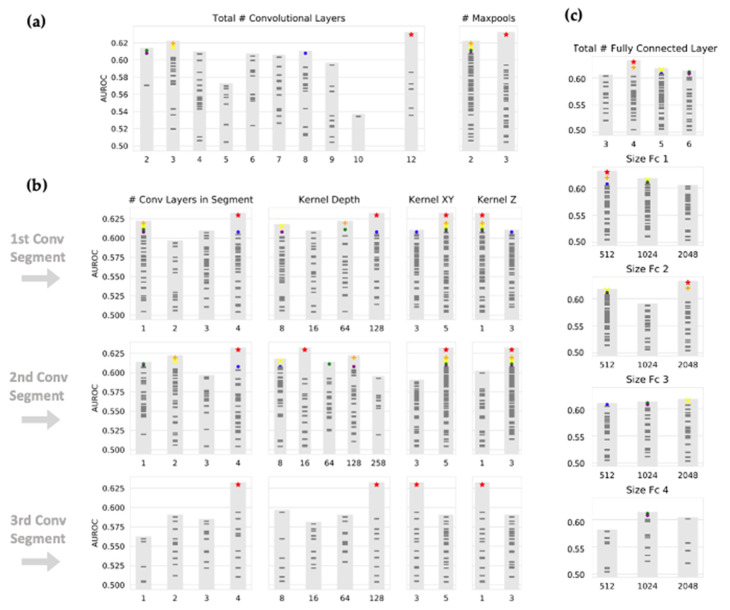
Selection of CNN architecture by Bayesian optimization. Neural network architecture was determined by Bayesian optimization. As in Figure 2, each mark represents a different set of hyperparameters. (**a**) The total number of convolutional layers and corresponding performance is shown. Convolutional layers are organized into ‘segments’ with interpolated maxpool layers, each segment composed of between 1 and 4 convolutional layers. (**b**) The average AUC for each hyperparameter within convolutional segments is shown. A preference for large and shallow kernels (5 × 1) is shown in lower levels, while large and deep kernels (5 × 3) seem to be preferred in higher convolutional layers. (**c**) The size and corresponding performance of fully connected layers are shown.

The best-performing CNN (depicted as red stars in Figure 3 and Figure 4) used masked T1w MPRAGE post-contrast, T1w post-contrast, and T2w FLAIR images as input. The network architecture consisted of three convolutional segments, each composed of four convolutional layers. The kernel sizes were 5 × 5 × 1, 5 × 5 × 3, and 3 × 3 × 1 for the first, second, and third convolutional segments, respectively. These were followed by three fully connected layers, with the number of neurons as follows: 512, 2048, 128.

The choice of input image modalities and the presence or absence of segmentation information had the most significant impact on model performance, with the highest performance seen for masked images and all imaging sequences. Figure 3 shows the objective function results (mean AUC over classes and four-fold validation) for all 100 combinations of hyperparameters, grouped by selection of masking type and input imaging sequence. The CNN architecture itself had a less definitive impact on the performance. As shown in Figure 4, large kernel sizes were generally preferred to smaller ones, with larger kernels (5 × 5 × 3) at intermediate or higher layers. Figure 4 also shows the objective function output grouped by parameters relating to convolutional and fully connected layers.

We compared the performance of this optimized CNN with that of two experienced radiologists. The CNN outperformed both radiologists for every assessed primary tumor entity: Radiologist 1 achieved an AUC of 0.55, 0.52, 0.45, and 0.51, while radiologist 2 achieved an AUC of 0.68, 0.55, 0.63, and 0.43 for melanoma, small cell lung cancer, breast cancer, and lung adenocarcinoma, respectively. Table 1 demonstrates performance metrics for each rater (Radiologist 1 (R1), Radiologist 2 (R2), CNN) including True-Positive- and True-Negative-Rates based on correctly identified tumors out of 22 total cases per tumor type.

**Figure 5:**
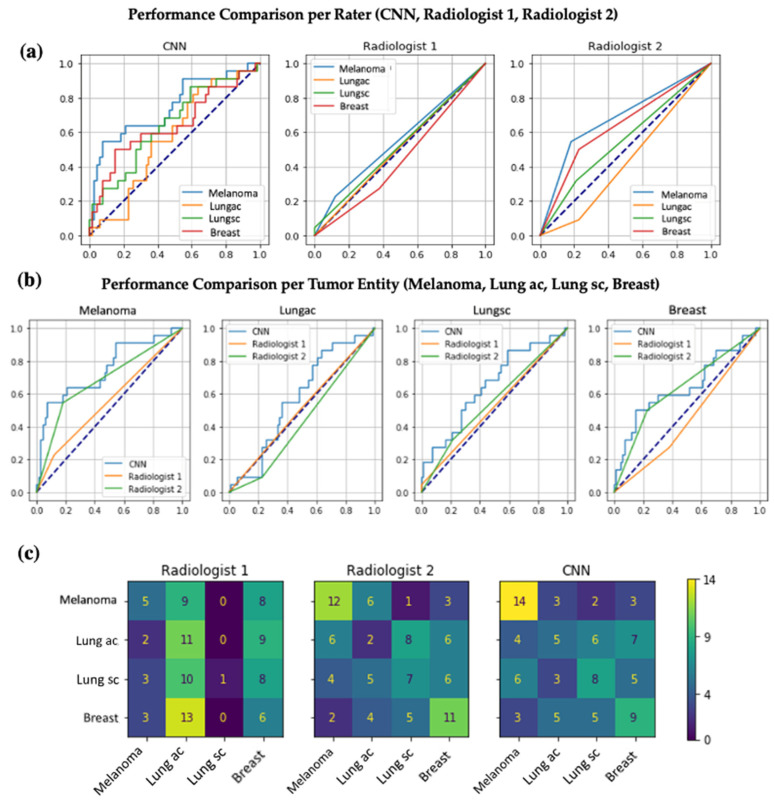
Performance comparison of the CNN and both radiologists using ROC curves. (**a**) All three panels show the ROC curves for the task of assigning brain metastases to the correct primary tumor (melanoma, lung adenocarcinoma, small cell lung cancer, breast cancer). The left panel shows an ROC curve for a CNN trained with all available training data using the optimal parameters as selected by Bayesian optimization on the test dataset. The middle and right panels are the ROC curves per tumor entity for two experienced radiologists (Radiologist 1 with an AUC of 0.55, 0.51, 0.52, 0.45 and Radiologist 2 with an AUC of 0.68, 0.43, 0.55, and 0.64 for, respectively, melanoma, lung adenocarcinoma, small cell lung cancer, breast cancer). (**b**) Each panel displays the direct comparison of the ROC curves of the CNN, Radiologist 1, and Radiologist 2 per included tumor entity. (**c**) Each panel shows a confusion matrix representing how many of the true labels on the Y-axis were predicted as the respective label on the X-axis. As an example, Radiologist 1 correctly assigned brain metastases to melanoma 5 times but more often incorrectly assigned metastases of lung adenocarcinoma or breast cancer to melanoma as the primary tumor origin (9 and 8 times, respectively).

**Figure 6:**
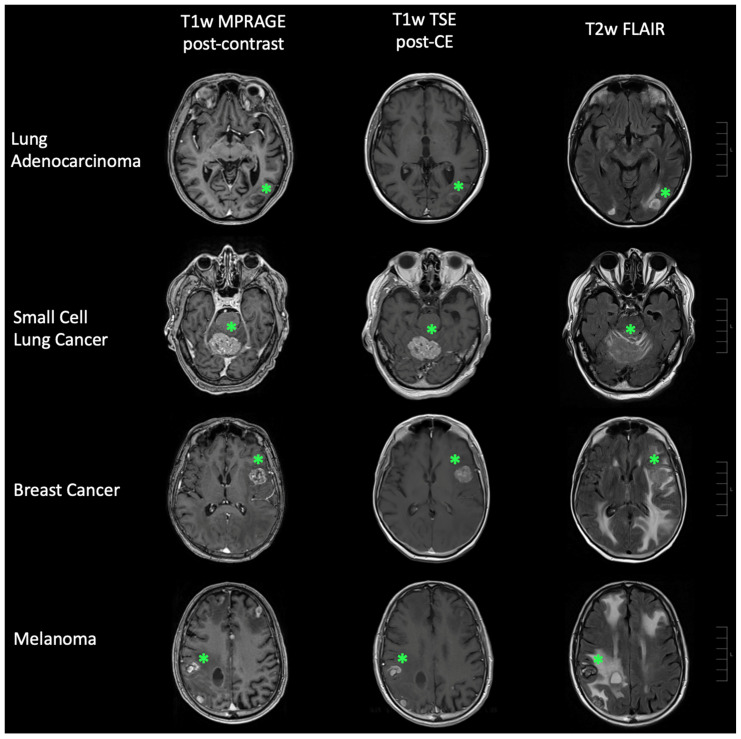
Representative cases of brain metastases. Brain metastases (green star) linked to lung adenocarcinoma, small cell lung cancer, breast cancer, and malignant melanoma are depicted. The left panel shows T1-weighted (T1w) MPRAGE post-contrast-enhancement (CE) imaging, the middle panel displays T1w TSE post-CE imaging, and the right panel features T2-weighted (T2w) FLAIR imaging. Scale bars (right side of each row) correspond to 10 mm per unit.

Figure 5 illustrates the performance comparison of the CNN and both radiologists using ROC curves. Figure 6 adds illustrating examples.

## 4. Discussion

In this study, we investigated the feasibility of using CNNs to determine the primary tumor of origin from MRI scans of brain metastases, with hyperparameters optimized using Bayesian optimization. The performance of the CNN was then compared with that of two expert radiologists who are extensively experienced in neuro-oncological imaging. Our results demonstrated that the highest model performance was achieved when using masked images with all imaging sequences—T1w MPRAGE and T1w TSE post-contrast as well as T2w FLAIR. Additionally, the network architecture appeared to have only minimal influence on the model’s performance. For certain tumor types, the CNN performed relatively well, particularly when compared to the diagnostic accuracy of the radiologists.

Currently, patients presenting with brain metastasis of unknown origin often undergo a time-consuming and invasive diagnostic process to locate the primary tumor. There is evidence to suggest that tumor identity can be inferred from radiographic information alone [17,18,19]. Previous studies have shown a correlation between location and number of metastases and the primary tumor origin. For example, lung cancer and melanoma tend to produce multiple intracranial metastases, whereas breast cancer typically results in solitary lesions [34,35]. Additionally, MRI imaging of melanoma is known for its high specificity, with T1w shortening and hypointensity on T2w STIR sequences correlating with melanin content in the tumor [36]. Several studies using radiomic features and machine learning models, such as random forests, had moderate success classifying brain tumors [17,18,19,37,38]. One study differentiated glioblastoma from metastatic brain tumors with an AUC of 0.8 [38], while another study classified five metastatic tumor entities, achieving moderate success with melanoma and non-small cell lung cancer, receiving an AUC of 0.82 and 0.64, respectively [17]. Our results indicate that, given more training data, MR images do indeed contain sufficient information to differentiate primary tumor entities.

CNN hyperparameters are typically set manually for segmentation and classification, which is time-consuming, potentially prone to errors, and, therefore, can lead to suboptimal hyperparameters [39]. By automating hyperparameter selection and model configuration, Bayesian optimization efficiently guides an optimized neural network setup. Bayesian optimization is known to find superior hyperparameter combinations with far fewer iterations than grid [20] or manual searches [40,41]. Recent studies have found that a Bayesian-optimized CNN can outperform manually tuned or standard models, reaching classification accuracies around 97–99% in brain tumor MRI tasks [39,40].

Interestingly, our findings suggest that neural network architecture, including the number of convolutional and fully connected layers, is not the most critical factor in determining performance. Our top-performing networks varied across different architectural configurations. However, it is noteworthy that larger kernel sizes are preferred in higher convolutional layers, as seen in Figure 4, where a 5 × 5 × 3 kernel outperforms a 3 × 3 × 1 kernel after the first max-pooling step. This is, to a certain degree, in contrast with the common practice in commercial applications, where smaller 3 × 3 convolutional kernels are typically favored. Given that convolutional kernels and iterative layers represent a ‘receptive field’, our findings suggest that larger receptive fields, enabled by larger kernel sizes, improve model performance.

While the relatively small size of medical datasets is often seen as a limitation, it offers some advantages in this context. Smaller datasets make use of SMBO techniques, such as Bayesian optimization, computationally feasible. With datasets numbering in the millions, training time would be intractable. Thus, with small datasets, it is possible to explore not only optimal neural network architectures but also the most relevant input data for model training. From our Bayesian optimization process, we observed that the T2w FLAIR data may not significantly benefit model performance, as demonstrated in Figure 3. This is somewhat counterintuitive, given the increasing attention to the role of the tumor microenvironment [5,42,43,44], which is thought to influence peritumoral edema [45,46] and other features detectable by T2w FLAIR. Despite this, our findings suggest that, at least for the current dataset, T2w FLAIR may not contribute as much useful information for differentiating primary tumor entities in brain metastases.

However, model performance could potentially improve if standardized MRI acquisition recommended by the Response Assessment in Neuro-Oncology-Brain Metastases (RANO-BM) working group were used. Kaufmann et al. have proposed a standardized MRI protocol for more consistent assessment of brain metastases at diagnosis and during treatment evaluation [47]. This protocol, based on RANO-BM [48] and the Brain Tumor Imaging Protocol (BTIP) [49], emphasizes the use of pre- and post-contrast 3D T1-weighted sequences, along with high-resolution T2w imaging, which, as for the latter, we did not include. The T2w FLAIR sequences we used are also recommended to detect vasogenic edema optimally [47]. Including additional T2w sequences may provide a more comprehensive representation of tumor characteristics and microenvironmental features, potentially enhancing model performance.

Moreover, certain tumor characteristics, such as the presence of blood in renal metastases or melanin in melanoma, may show specific imaging features. For instance, melanin in melanoma is known to produce paramagnetic artifacts on susceptibility-weighted imaging (SWI) and conventional T2*weighted sequences [50], which could aid in the classification of metastatic lesions. Therefore, the inclusion of additional imaging sequences, such as SWI, could potentially improve diagnostic performance by highlighting unique characteristics of certain tumors. Similarly, diffusion-weighted imaging (DWI) can reveal restricted diffusion in brain metastases due to increased cellularity, a feature often used to differentiate brain metastases from other intracranial lesions such as abscesses [8]. Although both brain metastases and abscesses may appear as ring-enhancing lesions on post-contrast T1w imaging, diffusion restriction is usually more pronounced in abscesses, particularly in the central non-enhancing portion [51,52,53,54]. However, it is important to note that approximately 4% of brain abscesses may present without diffusion restriction [54]. Including DWI in the imaging protocol could provide additional diagnostic information, further enhancing the performance of the CNN in distinguishing between various brain lesions.

This study has several limitations. As with most medically related studies, small dataset sizes limit the performance of neural network algorithms. In our case, the desire for equally sized classes meant that the smallest class, melanoma, constrained the size of all other datasets. Additionally, selecting an appropriate performance metric for the Bayesian optimization process is crucial and can be challenging. In this study, the AUC for each class was calculated and averaged together. The AUC was selected because it is widely used in the medical field to assess diagnostic accuracy. However, other metrics, such as the F1-score, could be considered in future studies despite criticisms that the F1-score may underrepresent false negatives. Another limitation of this study is that we did not perform secondary segmentations as a quality metric for the CNN segmentations, which makes it difficult to objectively evaluate the segmentation quality.

## 5. Conclusions

In this study, we demonstrate that Bayesian optimization can effectively select hyperparameters to improve model performance in classifying brain metastases by their primary tumor origin. Using this approach, we trained a CNN that performed moderately well in classifying melanoma and breast cancer but rather poorly at classifying lung adenocarcinoma. Overall, the CNN outperformed two expert neuro-oncological radiologists, each with extensive experience in brain tumor imaging. These findings suggest that with larger datasets and further refinement, neural networks could be capable of determining the primary tumor origin from imaging alone, leading to more efficient diagnostic and therapeutic decision-making.

## Figures and Tables

**Table 1:** Performance metrics for each rater per tumor entity.

		TP	FP	TN	FN	TPR (Sen)	TNR (Spec)	FPR	FNR
Melanoma	R1	5	8	58	17	22.7%	87.9%	12.1%	77.3%
	R2	12	12	54	10	54.5%	81.8%	18.2%	45.5%
	CNN	14	13	53	8	63.6%	80.3%	19.7%	36.4%
Lung ac	R1	11	32	34	11	50.0%	51.5%	48.5%	50.0%
	R2	2	15	51	20	9.1%	77.3%	22.7%	90.9%
	CNN	5	11	55	17	22.7%	83.3%	16.7%	77.3%
Lung sc	R1	1	0	66	21	4.5%	100.0%	0.0%	95.5%
	R2	7	14	52	15	31.8%	78.8%	21.2%	68.2%
	CNN	8	13	53	14	36.4%	80.3%	19.7%	63.6%
Breast Cancer	R1	6	25	41	16	27.3%	62.1%	37.9%	72.7%
	R2	7	14	52	15	31.8%	78.8%	21.2%	68.2%
	CNN	9	15	51	13	40.9%	77.3%	22.7%	59.1%

Legend: TP = True Positive, FP = False Positive, TN = True Negative, FN = False Negative, TPR = True-Positive-Rate, Sen = Sensitivity, TNR = True-Negative-Rate, Spec = Specificity, FPR = False-Positive-Rate, FNR = False-Negative-Rate; R1 = Radiologist 1, R2 = Radiologist 2; Lung ac = Lung adenocarcinoma; Lung sc = Small cell lung cancer.

## Data Availability

The data supporting the reported results are available upon reasonable request. However, due to privacy and ethical restrictions, data sharing will be conducted in strict compliance with applicable data protection regulations and institutional policies. Researchers interested in accessing the data may contact the corresponding author.

## Supplementary Materials

The following supporting information can be downloaded at https://www.mdpi.com/article/10.3390/brainsci15050450/s1, Text S1: Further algorithmic information. References [55,56] are cited in the Supplementary Materials.

## Abbreviations

The following abbreviations are used in this manuscript:

CNN	Convolutional neural network
MRI	Magnetic resonance imaging
CT	Computed tomography
AUC	Area-Under-the-Curve
ROC	Receiver Operating Characteristics
SD	Standard deviation
MPRAGE	Magnetization Prepared-RApid Gradient Echo
FLAIR	Fluid-Attenuated Inversion Recovery
STIR	Short-Tau-Inversion-Recovery-Sequenz
SWI	Susceptibility-weighted imaging
DWI	Diffusion-weighted imaging
CE	Contrast enhancement
AI	Artificial Intelligence
SMBO	Sequential model-based optimization
PACS	Picture Archiving and Communication System
MITK	Medical Imaging Interaction Toolkit
NIFTI	Neuroimaging Informatics Technology Initiative
DKFZ	German Cancer Research Center
MNI	Montreal Neurological Institute
RANO-BM	Response Assessment in Neuro-Oncology-Brain Metastases
