# Supplementary material for: Bayesian-Optimized Convolutional Neural Networks for Classifying Primary Tumor Origin of Brain Metastases from MRI
Source: Brain Sci. 2025 Apr 25;15(5):450. doi: 10.3390/brainsci15050450 (PMC12110443; doi:10.3390/brainsci15050450)
Supplement: Supplementary file 1 [file brainsci-15-00450-s001.zip › brainsci-3578370-supplementary.pdf]

Supplementary material

Supplementary Text S1 *Further algorithmic information*

SMBO methods consist of five parts, namely 1) a hyperparameter space to be searched, 2) the actual objective function that, given an instance of hyperparameters selected from the hyperparameter space, returns a performance measure for those hyperparameters, 3) a surrogate model of the objective function, 4) a criteria for selecting the next hyperparameters, and 5) a history of parameters already searched [1]. Various SMBO methods have been developed, differing in steps three and four, namely the model of the objective function (e.g., Gaussian Processes, Tree Parzen Estimators) and the criteria to select new parameters (e.g., Maximum Likelihood) [2]. The algorithm then alternates between two states: a 'searching' state, where unknown areas of the hyperparameter space are randomly explored and used to refine the surrogate model of the objective function, and an 'optimization' state, where high-performing areas of the hyperparameter space are explored in high density.

1. Dewancker I, McCourt M, Clark S. Bayesian Optimization for Machine Learning : A Practical Guidebook. arXiv:161204858 [cs]. 2016.

2. Lacoste A, Larochelle H, Laviolette F, Marchand M. Sequential Model-Based Ensemble Optimization. arXiv:14020796 [cs, stat]. 2014.
